# Hyperuricemia research progress in model construction and traditional Chinese medicine interventions

**DOI:** 10.3389/fphar.2024.1294755

**Published:** 2024-03-07

**Authors:** Hongyan Zhou, Jingyi Yang, Xiaoqing Yuan, Xinyu Song, Xingcai Zhang, Ting Cao, Jiayu Zhang

**Affiliations:** ^1^ Institute of Chinese Medicine, Binzhou Medical University, Yantai, Shandong, China; ^2^ School of Pharmacy, University of TCM, Jinan, Shandong, China; ^3^ World Tea Organization, Cambridge, MA, United States; ^4^ The First Affiliated Hospital, School of Medicine, Zhejiang University, Hangzhou, China

**Keywords:** hyperuricemia, traditional Chinese medicine, urate lowering, hyperuricemia models, efficacy evaluation

## Abstract

Hyperuricemia (HUA), a severe metabolic disease derived from purine metabolism disorder, will lead to abnormally increased serum uric acid (SUA) levels in the body. Studies have shown that HUA is highly related to gout, hypertension, diabetes, coronary heart disease, chronic kidney diseases, and so on. Traditional Chinese medicine (TCM) shows excellent results in treating HUA because of its unique advantages of multi-metabolites and multi-targets. This article reports on the use of TCM components for uric acid (UA)-lowering activity with excellent efficacy and low side effects based on established HUA models. This work summarizes the advantages and limitations of various HUA disease models for efficacy evaluation. Applications of TCM in HUA treatment have also been discussed in detail. This paper reveals recent research progress on HUA in constructing evaluation models and systematic TCM interventions. It will provide a scientific reference for establishing the HUA model and suggest future TCM-related HUA studies.

## Introduction

**FIGURE 1:**
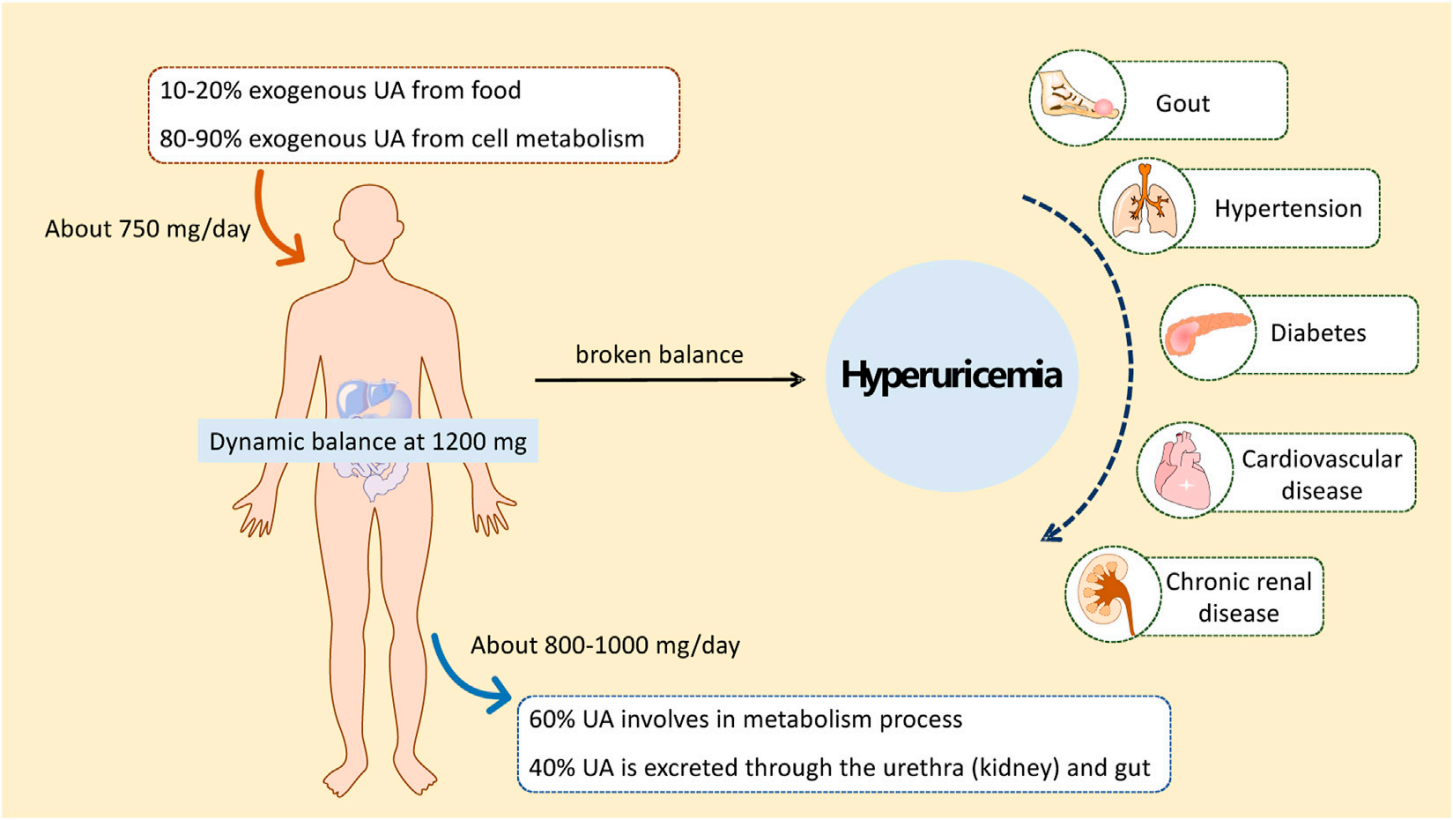
Occurrence of HUA and HUA-related diseases.

Hyperuricemia (HUA) is a metabolic disease with a high morbidity worldwide that is recognized as the “fourth leading cause of hypertension, hyperglycemia, and hyperlipidemia” (Zhang, 2015). HUA is diagnosed if the serum uric acid (SUA) concentration is higher than 417 μmol/L for men or 357 μmol/L for women (Jiang et al., 2020). Studies have shown that other diseases, such as gout, hypertension, diabetes, atherosclerosis, cardiovascular and cerebrovascular diseases, and kidney diseases, are associated with a high risk of long-term high uric acid (UA) status in the body (Figure 1) (Han et al., 2017; Kimura et al., 2021; Nishizawa et al., 2022; Kuwabara et al., 2023). Therefore, the study and treatment of HUA are of great practical significance for human health.

HUA is mainly treated by improving lifestyle or pharmacotherapy to reduce SUA levels (Alvarez-Lario and Alonso-Valdivielso, 2014). In terms of improving lifestyle, the intake of low-purine foods, stopping smoking and drinking alcohol, exercising, and controlling weight have proven to be effective measures for attenuating HUA (Morgan and Singh, 2021). In pharmacotherapy, UA-lowering drugs are classified into three categories: drugs that inhibit UA production (allopurinol, febuxostat, *etc.*), drugs that promote UA excretion (probenecid, benzbromarone, *etc.*), and drugs that promote UA decomposition (pegloticase, rasburicase, *etc.*) (Pacher et al., 2006; Terkeltaub, 2006; Cortes et al., 2010). Although these drugs can somewhat treat HUA, they have unavoidable side effects and clinical limitations (Saag et al., 2022). Therefore, developing safer and more effective therapeutic strategies for HUA is necessary (Flores et al., 2023).

Traditional Chinese medicine (TCM) refers to traditional medicine developed in China for thousands of years, containing natural or processed plants, animals, and minerals from nature. It is used for clinical therapy with the guidance of TCM theory and modern science (Ji et al., 2021; Cao et al., 2023). Compared to Eastern medicine, TCM has high safety characteristics, multiple metabolites, and multiple targets, indicating that it is suitable for HUA- and UA-level regulation. This review summarizes the pathogenesis, evaluation model of drug efficacy, and intervention treatment progress for HUA using TCM.

### Pathogenesis of HUA

**FIGURE 2:**
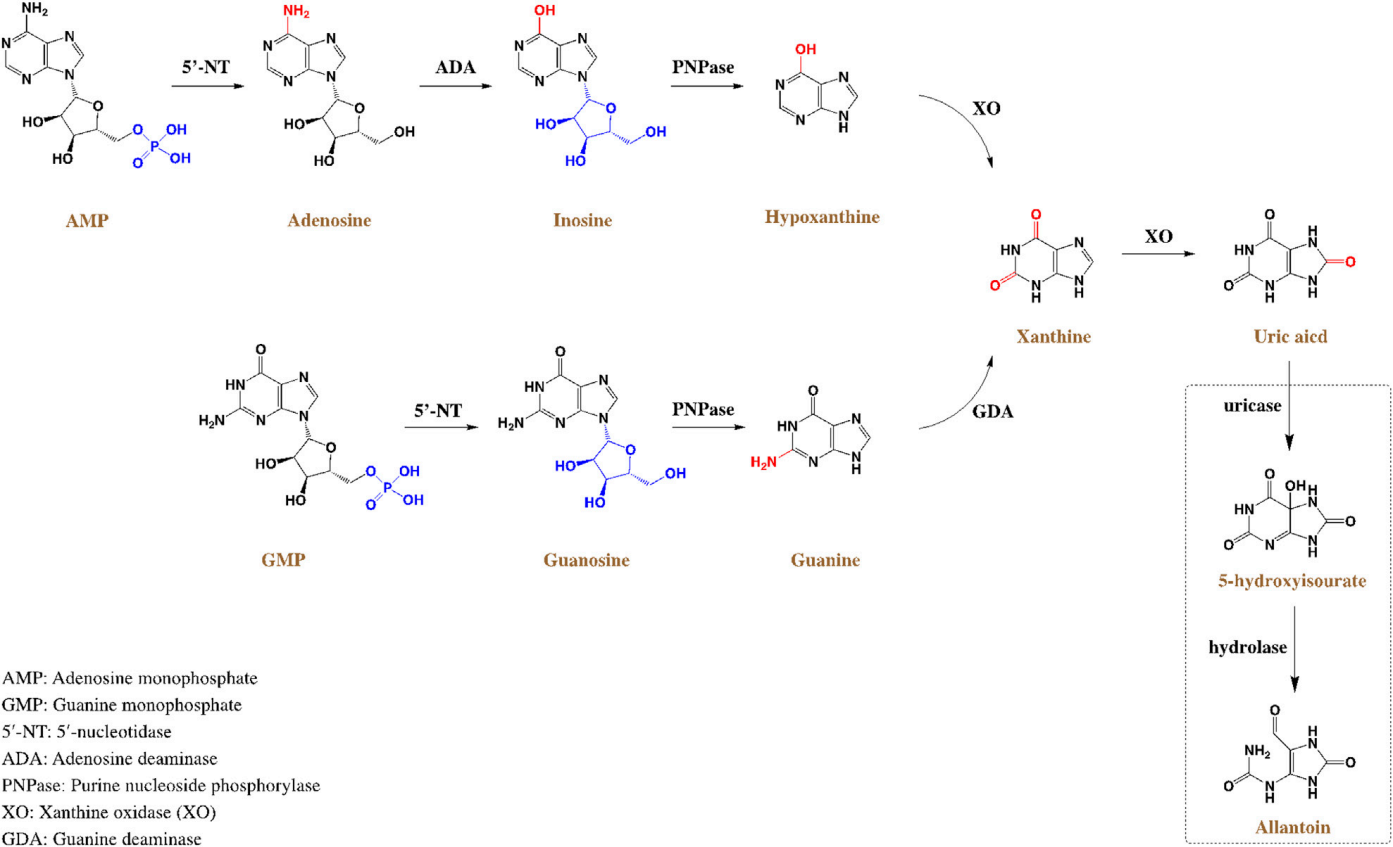
Schematic diagram of UA metabolism and generation process in the human body.

**FIGURE 3:**
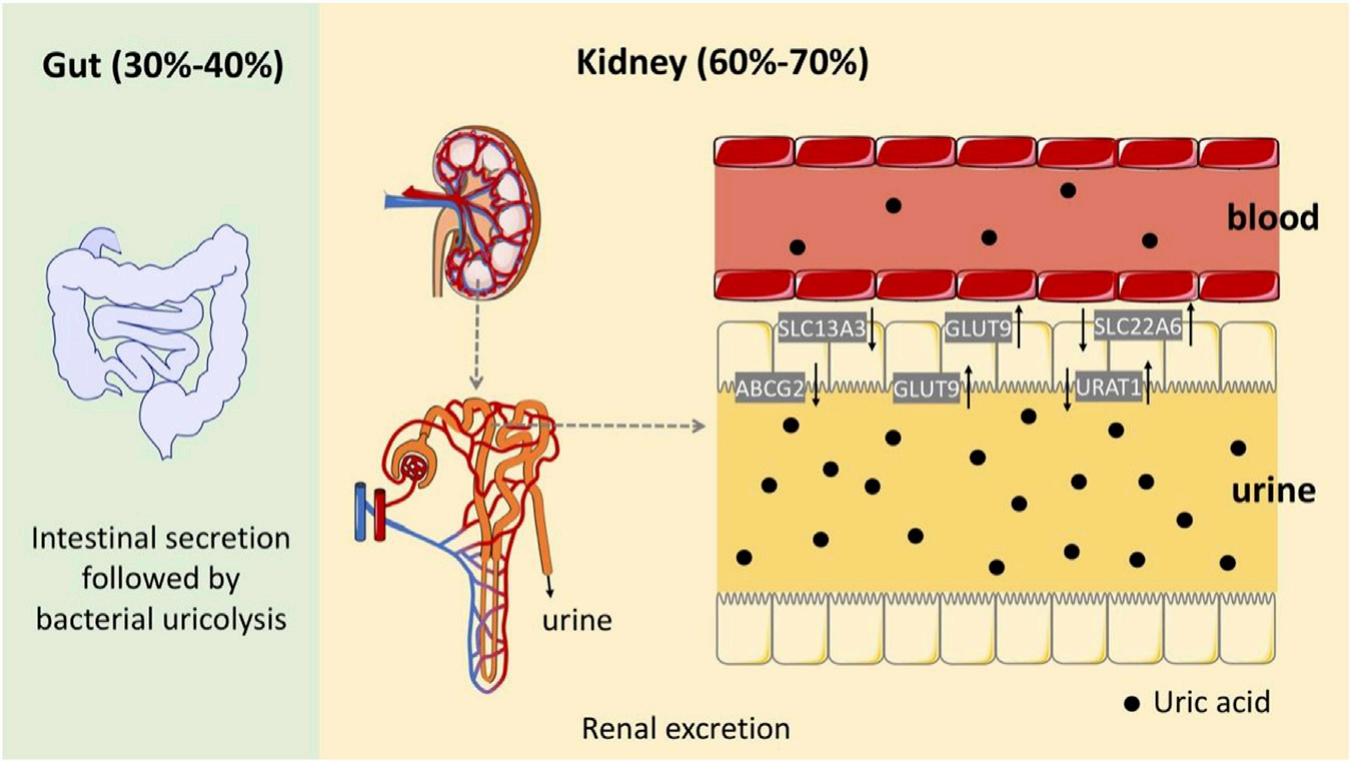
Schematic diagram of the UA excretion process in the human body through the intestines and kidneys.

HUA results from an imbalance of SUA in the human body, which results from excessive production or impaired renal excretion of UA during purine metabolism. UA is the end product of purine metabolism in the human body and is mainly synthesized in the liver, intestines, and other tissues (Lima et al., 2015). Two typical biosynthesis pathways of UA from purines are shown in Figure 2 (Mandal and Mount, 2015): 1) adenosine monophosphate (AMP) is degraded into adenosine and phosphates with the catalysis of 5′-nucleotidase (5′-NT). Subsequently, adenosine is deaminated by adenosine deaminase (ADA) to form inosine, which is subsequently transformed into hypoxanthine by purine nucleoside phosphorylase (PNPase). Finally, hypoxanthine is transformed into xanthine and then into UA by xanthine oxidase (XO). 2) Guanosine monophosphate (GMP) is dephosphorylated to form guanosine by 5′-NT, which is then transformed into guanine via PNPas catalysis. Guanine is converted into xanthine by guanine deaminase (GDA), which is further converted into UA via XO catalysis. UA exists in the body in an anionic state and cannot autonomously cross the cell membranes. In most mammals, including rats and mice, UA can be enzymatically converted to allantoin, which is more soluble and easily excreted in the urine (shown in the empty box in Figure 2). Approximately two-thirds of UA is excreted from the body through the kidneys, while one-third is excreted through the gut (Fathallah-Shaykh and Cramer, 2014). UA mainly depends on UA transporters to complete its reabsorption and excretion, which include influx and efflux transporters to maintain UA homeostasis, such as SLC and ABC transporters expressed in human renal proximal tubule cells (Figure 3) (Ichida et al., 2012). The abnormal expression of UA transporters results in the accumulation of UA in the body and the occurrence of HUA.

### Understanding of HUA in Chinese medicine

The symptoms of HUA are assigned to “*gout* ,” “*lijie*,” “*rebi*,” and so on in the TCM field. Skilled TCM practitioners believe that HUA is caused by inborn weakness and kidney qi deficiency, six exogenous pathogenic factors, internal damage because of seven emotions, emotional frustration, dietary intemperance, etc. (Feng et al., 2022). The pathogenesis of HUA includes kidney qi deficiency, zang–fu disorders, visceral dysfunction, phlegm, and blood stasis (Jianmin et al., 2022). This qi cannot push the flow of body fluids and blood, resulting in endogenous congestion because of the deficiency of the liver and kidneys. HUA results from a deficiency in origin and excess superficiality and is diagnosed according to the differentiation of zang and fu, mainly in the spleen and kidneys. In short, HUA is closely related to the five zang–fu organs, especially the spleen, liver, and kidneys.

An important function of the spleen is to transport and transform nutrients into the human body (Yang et al., 2020; Huang et al., 2022; Jin et al., 2022; Chen et al., 2023). If the spleen runs unhealthily, dampness and turbidity are endogenously generated, and dampness, heat and phlegm turbidity accumulate, leading to excessive UA production in the blood (Wang et al., 2023). The liver controls the conveyance and dispersion. If liver function is disrupted, the kidneys become dysfunctional. Dampness-heat and phlegm turbidity decrease, reducing UA excretion in the blood (Li and Bao, 2020). Both excessive UA production and reduced UA excretion can form HUA. According to TCM theory, HUA results from dampness, phlegm, and blood stasis because of an intemperate diet or liver–kidney qi deficiency. Clinically, the symptoms of HUA can be classified into a phlegm-dampness block, yang qi deficiency, yin blood deficiency, dampness-heat accumulation, qi stagnation and blood stasis, cold condensation and qi junction, and so on (Zhang et al., 2022a), which can be regulated and treated by tonifying the kidney and spleen, tonifying the liver and kidney, eliminating dampness and turbidity, clearing dampness and heat, promoting blood circulation, removing blood stasis, and so on.

## Research modes of HUA for pharmacodynamic evaluation

### Cell models

The HUA cell model has the advantages of simple operation, small dosage, and high screening efficiency. Establishing an efficient and stable cell model for studying HUA will be conducive to screening UA-lowering drugs and the corresponding mechanics studies. UA is usually produced in the liver and excreted through the kidneys and intestines. Thus, kidney, liver, and intestinal cell lines are primarily used to construct HUA cell models (Table 1). The most commonly used cell lines include human proximal convoluted human kidney-2 (HK-2 cells), human tubular epithelial cells (HKC), human embryonic kidney cells 293 (HEK293 cells), human normal liver cells (LO2 cells), human liver cancer cells (HepG2 cells), and human colorectal adenocarcinoma cells (Caco-2 cells) (Wu et al., 2021a).

**TABLE 1:** Published cell lines for the establishment of HUA models.

Organ	Cell type	HUA inducer	Model evaluation	Reference
Liver	Human normal liver cells (LO2)	Adenosine (2.5 mmol/L) + XO (final 0.005 U/mL)	Rapidly rising uric acid levels; short maintenance time	Liu et al. (2017)
	Buffalo rat liver-3A (BRL3A) cell	Xanthine (4 μmol/L)	Rapidly rising uric acid levels	Adachi et al. (2017)
	Primary hepatocyte	Xanthine (200 μM) + potassium oxazate (500 μM) + bisphenol A (0.1 μM or 1 μM)	Stable; rapidly rising uric acid levels	Ma et al. (2018)
Kidney	Human embryonic kidney 239 (HEK293) cell	Torasemil	Stable and reliable; screening and studying drugs acting on hOAT4	Hagos et al. (2007)
	Madin–Darby canine kidney (MDCK) cell	Lentivirus infection	Screening and studying drugs acting on hOAT4	Chen et al. (2016b)
	Renal tubular epithelial cell (RTEC)	UA (1,500 μmol/L)	Simple model method; complex primary cell culture process	Zang et al. (2011)
	Human kidney-2 (HK-2) cell	Fructose (5 and 10 mmol/L)	Large-scale drug screening	Wang et al. (2018b)
Intestine	Human intestinal epithelial cells (Caco-2)	UA (500 mg/L)	Hyperuricemic intestinal injury model	Glushakova et al. (2008)
	Rat small intestine crypt epithelial cells (IEC-6)	UA (100 mg/L)	Hyperuricemic intestinal injury model	Li et al. (2023)
Vessel	Human umbilicus vein endothelial cell (HUVEC)	UA (1,500 μmol/L or 20 mg/)	Hyperuricemic vascular endothelial injury model	Hong et al. (2020)
	Vascular smooth muscle cell (VSMC)	Urate (5 mg/dL)	Hyperuricemic vascular injury model	Yan et al. (2019)

### Increased UA production

#### UA as an inducer

The HUA research model of HUA is established by directly adding UA or urate to the culture medium when culturing the cells. For example, Zang et al. (2011) added 1,500 μmol/L UA into the culture medium to mimic the excessive UA intake of renal proximal tubular epithelial cells (RTECs). The result showed that the UA absorption rate reached its maximum at 30 min and plateaued after 60 min. The cells were then maintained in a high-UA environment to establish the HUA research model. This research also proved that the absorption process of UA is closely related to URAT1, which could be used for pharmacodynamic screening and molecular mechanism research. Similarly, Yan et al. (2019) incubated osteoblasts and vascular smooth muscle cells (VSMCs) with 5 mg/dL urate crystals for 48 h to establish an HUA model. Therefore, UA can be a common and direct inducer for developing HUA cell models.

#### Adenosine as an inducer

Increasing adenosine concentration promotes UA synthesis (Figure 2). Liu et al. (2017) used LO2 cells as target cells and adenine and adenosine as inducers to construct an *in vitro* HUA model. The cells were treated with 2.5 mmol/L adenosine and 0.005 U/mg XO for the final production of UA. Hou et al. (2019) used HK-2 cells and adenosine to construct an HUA research model using XO. XO, an important enzyme involved in UA production in human cells, can be used as a drug target in UA-lowering strategies. The level of UA in the cell supernatant can be measured by HPLC, significantly realizing the real-time monitoring of the UA concentration. Cell models of HUA established using this method require small doses of reagents and have low experimental costs. They can also be used to screen UA-lowering drugs. However, these cells are mostly immortal cell lines, a potential limitation because of the different epigenetics of natural cells in the human body.

#### Xanthine as an inducer

Hypoxanthine and xanthine are the precursors of UA during purine metabolism (Figure 2). XO can oxidize hypoxanthine to xanthine and subsequently oxidizes xanthine into UA. Adachi et al. (2017) established a cell model of HUA by stimulating BRL3A cells with 4 mol/L xanthine for 48 h. A higher concentration of UA was observed than that in the control experiment by monitoring XO activity and UA levels in the cell supernatant. Based on these results, an *in vitro* HUA cell model was developed using xanthine as an inducer.

#### Bisphenol A as an inducer

Bisphenol A can increase the reaction efficiency of transforming xanthine to UA by enhancing the activity of XO, giving the possibility of high UA with the presence of bisphenol A. For example, Ma et al. (2018) incubated primary mouse liver cells and 200 M xanthine for hours. After the intervention of 24 h with 0.1 μM or 1 μM bisphenol A, the synthesis of UA in primary hepatocytes was significantly increased. During the experiment, it was necessary to add 500 μM potassium oxonate (PO) to inhibit the decomposition of UA by uricase because of the presence of uricase in primary hepatocytes. This study used primary cells for modeling; however, primary cells have several intrinsic shortcomings, including a difficult separation process, a high proportion of heterozygotic cells, and a highly differentiated tendency.

### Decreased UA excretion

Human organic anion transporters OAT1 (hOAT1) and OAT3 (hOAT3) are expressed on the basolateral side of proximal tubule cells, facilitating the uptake of organic anions into the cell. The rate absorption transporter hOAT4 can reabsorb urate from the renal tubule into the blood. Hagos et al. (2007) modified HEK293 cells to stably express hOAT1, hOAT3, and OAT4. After incubation with torasemide, hOAT4 expression increased, and simultaneously, the activities of hOAT1 and hOAT3 were inhibited, leading to the inhibition of urate excretion and the occurrence of HUA.

An artificial HUA cell model, established by increasing the expression of human URAT1 (hURAT1), promoted UA reabsorption and inhibited UA excretion. hURAT1 reabsorbs UA into epithelial cells and re-enters blood circulation. For example, Chen et al. (2016a) generated Madin–Darby canine kidney (MDCK) cell lines that persistently expressed hURAT1 *in vitro*. This cell line (MDCK-hURAT1) significantly enhanced the ability of cells to transport UA, making it suitable for screening urate-lowering drugs.

### Others

Among the inducers of HUA, fructose is a special substance that can both promote the synthesis of UA and interfere with the excretion of UA, leading to an increase in SUA content (Perez-Pozo et al., 2010). Fructose is phosphorylated by fructokinase to produce adenosine diphosphate (ADP) in the cytoplasm, which is then metabolized to UA (Figure 2). For example, Chen et al. (2016b) and Wang et al. (2018a) added 5 mmol/L and 10 mmol/L fructose to the medium and incubated the fructose with HK-2 cells for 48 h and 72 h. Compared to the control group, the concentration of UA in cells treated with fructose increased significantly. In addition to the increase in UA, fructose can promote cell inflammation and kidney damage and decrease the clearance efficiency of UA. *In vitro* studies confirmed that fructose stimulates the expression of monocyte chemoattractant protein (MCP-1) and upregulates the expression of the endothelial pro-inflammatory mediator intercellular adhesion molecule (ICAM-1) in HK-2 cells, which promotes inflammatory response and inhibits UA excretion (Glushakova et al., 2008). HK-2 cells are proximal convoluted tubule cells derived from normal kidneys that were immortalized by incorporating the HPV-16 E6/E7 gene. Thus, these cells have the advantages of immortality, a lack of ethical controversy, and reliable reproducibility. This method provides a pathway for establishing cell models of HUA and a reference for the large-scale screening of UA-lowering drugs *in vitro*.

## Animal models

### Selection of model animals

**FIGURE 4:**
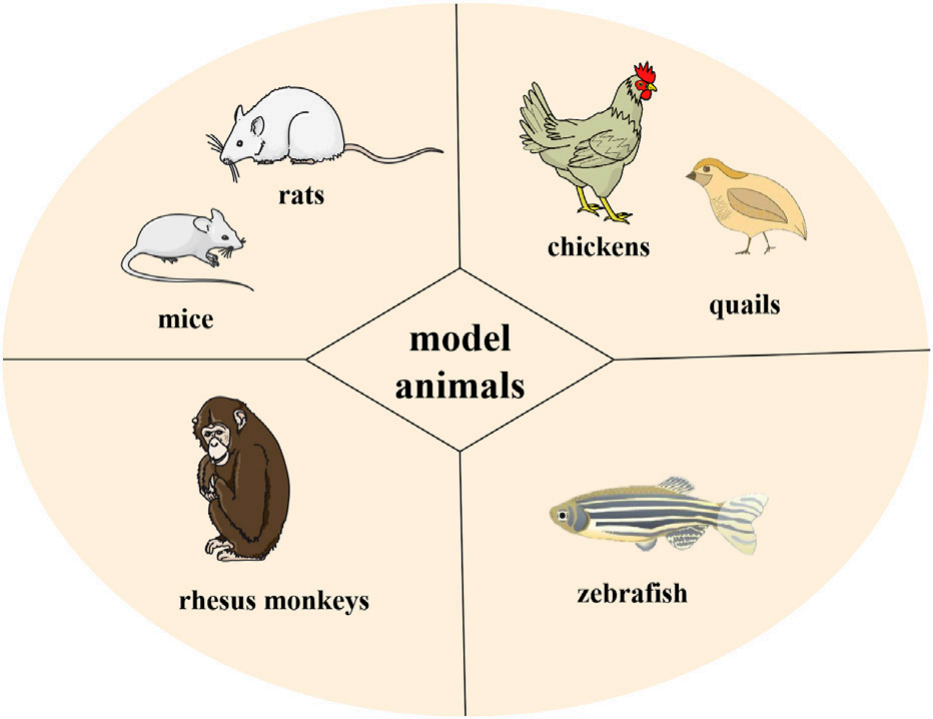
Model animals used in HUA research.

The animal models used in HUA research mainly include poultry species, rodents, and primates (Figure 4). Chickens and quails are common model animals used in HUA studies because they have the same UA metabolic pathways as humans. For example, Bian et al. (2020) successfully used quail to establish a research model for HUA by feeding them yeast solutions. However, divergence between poultry species and humans limits their application in HUA studies because of differences in other metabolic processes.

Rodents, especially rats and mice, are the most commonly used animal models in the laboratory. They are more physiologically similar to humans than poultry species. Moreover, they have a smaller body size and are easier to feed than other laboratory animals. However, rodents have urate oxidase in their bodies, which can convert UA into a more soluble allantoin that is excreted from the body. This enzyme activity indicates that rodents cannot maintain a stable HUA status for a prolonged period without external interference.

Primates, with physiological processes similar to humans, have almost the same UA metabolic pathway and can theoretically be considered perfect animal models for HUA studies. However, because of their high cost, tedious operation, and specific metabolic differences, only a few studies have adopted primates as animal models for HUA research.

Zebrafish, as a vertebrate model, have recently become a new animal model for studying human diseases and drug therapies, with the advantages of low price, small size, and short experimental period. However, species differences still exist between zebrafish and humans, and zebrafish-based HUA research models are still in the preliminary exploration stage (Zhang et al., 2019).

No ideal animal model can substitute for human beings in HUA studies. However, as common laboratory animals, mice and rats still play a vital role in studying HUA despite urate oxidase in the body, especially regarding HUA formation and organ damage. Therefore, we will emphasize recent HUA studies using mice or rats as animal models in the section on animal studies.

### Increased UA production

#### UA as an inducer

Animal models can be established by gavage or intraperitoneal injection of UA. For example, Chen et al. (2001) administered an intraperitoneal injection of 250 mg/kg UA and obtained a good HUA disease model. SUA levels in mice peaked 10 min after injection, were maintained for 4 h, and then returned to baseline after 5 h. This method can be used as a fast HUA study tool, such as the selection of UA-lowering drugs, but it is unsuitable for an in-depth study of the specific mechanism of HUA disease.

#### Adenine as an inducer

Adenine can be converted into AMP by adenine phosphoribosyltransferase (APRT), which simultaneously increases the activity of XO, promoting synthesis UA as mentioned above (Figure 2). Yang et al. (2011) intragastrically administered adenine (200 mg/kg, in sodium carboxymethyl cellulose) consecutively for 30 days to establish a rat model of HUA. After 10 days, the SUA level of the rats increased significantly, and the rats showed symptoms of emaciation, kidney injury (kidneys were covered with greyish-yellow particles), and severe renal tubule injury. In short, adenine significantly increased SUA levels and induced HUA in a rat model. The model has good stability, but the accompanying organ injuries must be considered.

#### Hypoxanthine as an inducer

Hypoxanthine metabolizes into xanthine via XO catalysis, which eventually generates UA. Zheng and Huang (2011) established an HUA mouse model by intraperitoneal injection of hypoxanthine (1,000 mg/kg). The results showed that the UA level in the mice dramatically increased 30 min after injection but significantly decreased 1 h later. The duration of high UA levels induced by this method is efficient but short, making it suitable for establishing animal models of acute HUA.

#### Yeast as an inducer

Yeast can increase the activity of XO, which promotes the production of UA, disorders of purine metabolism, and, ultimately, leads to the occurrence of HUA. Chen et al. (2003) attempted to add different concentrations of yeast paste to mice by intragastric administration, and the results showed that the UA levels of mice increased continuously when the feed dosage of yeast paste was 15–30 g/kg for 1 to 2 consecutive weeks. A higher concentration of 60 g/kg yeast paste led to the death of the mice. This finding is consistent with previously published experimental phenomena, which indicated that yeast powder could sometimes cause rats to exhibit severe weight loss and poor physical condition. In summary, yeast, as an inducer of HUA in animals, was concentration-dependent and was always added with other induction drugs for disease modeling. Therefore, using yeast as an inducer of HUA is feasible, but a detailed experimental design needs to be explored.

### Decreased UA excretion

The antituberculosis drug ethambutol competitively inhibits the excretion of UA in the kidney, leading to an increase in SUA levels. Ethambutol and niacin alone are rarely used as modeling agents to establish HUA models. These drugs are often administered in combination with other drugs. In principle, the HUA model induced by anti-TB drugs is identified as secondary HUA, which is not suitable for disease modeling of primary HUA in animals.

### Inhibit urate oxidase activity

Potassium oxazinate (PO) is a drug commonly used to model HUA in mice and rats. It has a purine-analogous structure and can bind to uricase to inhibit UA oxidase activity, leading to the accumulation of UA in the animal body. Wu et al. (2021b) established a mouse model of HUA by intraperitoneal injection of PO for 7 days. The UA level was higher in the model group than in the control group, indicating that the model construction of HUA was successfully achieved. In addition, Wang et al. (2018b) established a rat model of HUA by intraperitoneal injection of PO into Wistar rats. The results showed that 300 mg/kg was the optimal concentration of PO that could maintain clinically defined HUA concentrations for 2 h. The PO-induced animal model of HUA has the advantages of simplicity, convenience, and sensitivity and is suitable for establishing short-term acute animal models of HUA. However, PO inhibits the activity of UA oxidase and other enzymes, such as orotate phosphoribosyl transferase, which sometimes results in uncertain interference with experimental results.

### Combination modeling

Combination modeling combines several metabolites as inducers for HUA modeling (as mentioned above in the section Yeast as an inducer). A combination of substances is the most common situation, such as a combination of two UA precursors, drugs that decrease UA excretion, and drugs that inhibit uricase activity. This method has the advantages of synergistically increasing the SUA level, elongating the maintenance time of the high SUA level, increasing the stability of the model, and reducing kidney damage. Thus, it is popular in animal HUA modeling.

### Combination of adenine and ethambutol

Shen et al. (2019) used 100 mg/kg adenine and 250 mg/kg ethambutol administered by intragastric administration to prepare a rat model of HUA. They investigated the mechanism of *Dendrobium ferruginosa* Four Myriad Formula in lowering UA. The UA level of the model group increased gradually after 1, 2, and 3 weeks compared with that of the control group, indicating that the rat HUA model was successfully established. Unfortunately, the rats showed symptoms of kidney injury, such as glomerular atrophy. Therefore, despite its ability to induce animal models, the combination of adenine and ethambutol has a limited application range because of organ damage.

### Combination of PO and hypoxanthine

Studies have shown that combining PO and hypoxanthine has the advantages of a fast response and long maintenance time for high SUA levels. He et al. (2021) established a mouse model of HUA by intragastrically administering different concentrations of hypoxanthine and PO to KM male mice consecutively for 7 days. The results showed that the combination of 500 mg/kg hypoxanthine and 1,000 mg/kg PO was an appropriate experimental condition for successfully establishing a mouse model for HUA studies. The treated mice displayed various symptoms, including poor physical conditions, reduced diet, and mental malaise.

### Combination of PO and adenine

Li et al. (2021a) administered a combination of PO and adenine to SD rats intragastrically consecutively for 5 days to investigate the optimal experimental conditions for HUA modeling. After 1 week, the SUA level in the model group increased significantly compared to that in the control group, and an optimized result was achieved at a dosage of 1,500 mg/kg PO and 50 mg/kg adenine. This approach promoted the synthesis of UA and effectively inhibited the activity of uricase, which resulted in a rapid increase in UA levels in a short time. However, kidney injury caused by adenine nephrotoxicity must be considered when adenine is used to establish animal models for HUA studies.

#### Combination of PO and yeast

Di (2013) fed male SD rats different doses of yeast paste and PO by intragastric administration and intraperitoneal injection to establish a chronic HUA model. SUA levels in the rats were significantly increased, and the glomeruli, interstitia, and tubules displayed tiny pathological lesions after 28 days. Urate crystal deposition has also been observed in certain animals. SUA levels increased rapidly and were maintained for a long time when yeast paste was combined with 200 mg/kg PO extract. In addition, the degree of renal injury was low, and almost no rats died during the experiment. This approach also affects the heart, large and small arteries, and other tissues and organs, satisfying the symptoms of caused secondary HUA. This method provides an excellent drug screening platform for HUA in animals and a perfect pathway for studying the interaction between HUA and cardiovascular disease.

#### Combination of PO and fructose

Liu et al. (2018) created an animal model of HUA by administering subcutaneous injections of PO (100 mg/kg) and fructose (5% and 10%). The results showed that PO and fructose increased SUA levels quickly and maintained them for some time, indicating that the HUA model was successfully established. The combination of PO and fructose can be used to quickly establish an animal model of HUA, with the advantages of higher efficiency, greater stability, and longer sustainability than a single substance. PO combined with 5% fructose did not cause kidney damage and could be used to establish an animal model of asymptomatic HUA in the human body for mechanistic investigations and drug therapies. PO combined with 10% fructose caused obvious renal pathological lesions, which were an indicative of chronic kidney injury in the HUA study.

#### Combination of yeast and fructose

Bie (2017) used a 0.2 (w/w) yeast diet and 10% fructose to induce HUA in a rat model. The results showed that the SUA concentration remained constant at 2–4 weeks. Compared to yeast and PO, yeast and fructose induced a rapid and continuous increase in SUA levels and reduced kidney damage simultaneously. The combination of yeast and fructose is simple and inexpensive and can be used to establish a stable and reliable animal model of HUA.

### Others

Excessive fructose levels lead to elevated SUA levels. Excessive fructose can be phosphorylated by fructokinase to increase the level of AMP and the activity of XO, which both result in a higher UA concentration in the serum. However, excessive fructose will cause kidney injury and disrupt the UA transport system, inhibiting UA excretion and promoting UA reabsorption. Therefore, fructose increased UA production and decreased UA excretion. Li et al. fed SD rats 10% fructose daily and found that SUA levels dramatically increased from 20 to 40 days. Simultaneously, the expression of GLUT9 protein was upregulated, strengthening the UA reabsorption process by the renal tubules. Essawy et al. (2014) continuously fed rats with 60% fructose and found that the levels of both SUA and insulin increased significantly after 4 weeks. Based on these experiments, we speculate that fructose-induced animal modeling of HUA is more suitable for studying HUA accompanied by multiple complications such as diabetes or hyperlipidemia.

#### Genetic engineering for the modeling of HUA

Genetic engineering for modeling HUA refers mainly to the deletion of urate oxidase or urate transporter genes to better mimic the pathogenesis of HUA in the human body.

#### Urate oxidase gene knockout

The urate oxidase gene encodes uricase, which is not expressed in humans but is highly expressed in mice. Uricase catalyzes the conversion of UA into allantoin, a substance easily excreted in urine (Figure 2). Knocking out the urate oxidase gene in rats or mice can eliminate the disturbance in allantoin caused by urate oxidase and further simulate UA metabolism in the human body. Lu et al. (2018) knocked out the urate oxidase gene in C57BL/6J mice and found that approximately 40% of the mice survived for 62 weeks. All surviving mice had high SUA levels, indicating the possibility of modeling e HUA. However, mice must be raised in a sterile environment for this type of experiment model, and the mortality of the model animals must be considered in practice.

#### GLUT9 gene knockout

GLUT9, a type of urate transporter, is expressed in the human kidneys and is responsible for UA reabsorption. However, GLUT9 is mainly expressed in the liver of mice. Studies have demonstrated that while systemic GLUT9 gene knockout leads to high mortality in mouse embryos, hepato-specific GLUT9 gene knockout can result in high survival and UA levels in mice (Zhang et al., 2022b).

#### ABCG2 gene knockout

ABCG2 is a UA transporter involved in UA secretion in the renal tubules and small intestine. Takada et al. (Faulds et al., 2012) demonstrated that ABCG2 knockout mice had higher SUA levels than those in the control group. In addition, renal UA excretion increased, whereas intestinal UA excretion decreased.

Laboratory animals cannot completely recapitulate the physiology and pathology of UA metabolism in humans because of the species divergence between humans and other animals. Therefore, developing novel, simple, and effective animal models is crucial for investigating HUA.

### Animal models of HUA in the TCM field

Lin (2022) intragastrically administered 37.5 mg/kg adenine and 37.5 mg/kg PO to male C57BL/6 mice once a day for 3 weeks to construct a mouse model of HUA with kidney yang deficiency. The treated mice showed reduced weight, increased water intake, increased urine excretion, clear urine, difficult defecation, reduced grip strength, vertical fur, arching back, huddling, and weakened irritability. These symptoms indicated that the model of HUA with kidney yang deficiency was successfully established and could be used in subsequent mechanism exploration or drug screening.

Zhan et al. (Zhan, 2018) successfully established a rat model of damp-heat HUA using a high-fat and high-sugar diet, 10 mL/kg liquor gavage, and 200 g/L honey water *ad libitum* for 14 days. From the eighth day, the rats were fed a mixture of yeast extract (20 g/kg) and a high-fat diet and intraperitoneally injected with 300 mg/kg PO. Simultaneously, from the 10th day, the rats were kept in an artificial climate chamber with humidity and heat (relative humidity 89%–95%, temperature 30°C–34°C) for 4 days. The UA levels of the model group were significantly higher than those of the control group. In addition, the model group was tired and curled up with reduced activity, yellowed hair, and messy.

A damp-heat rat model of HUA was constructed by feeding rats fat and sweet food (5% lard, 3% egg yolk, and 5% honey added to the fodder) and administering *Escherichia coli* endotoxin (2 μg/kg) intravenously twice weekly for 6 weeks. The damp-heat HUA rat model could also be established by hypodermic injection of PO (200 mg/kg) and intragastric administration of ethambuterol (250 mg/kg) once daily for 6 weeks (Tang and He, 2019).

A rat model of HUA with spleen deficiency was established by feeding rats PO and high-purine supplements in a small-cage environment (Shang et al., 2020). High-purine supplements were administered during the entire process, and PO was intraperitoneally injected from the third week. Four weeks in total were required for the successful modeling of HUA with spleen deficiency. From the second week, the treated rats showed symptoms of reduced activity, yellow and less luster fur, reduced diet, and soft stools.

Shi et al. (2016) used a high-fat diet, hypoxanthine (500 mg/kg), and PO (100 mg/kg) to induce phlegm-dampness in a rat model of HUA. The rats were treated once daily for 30 days. During this process, the rats showed symptoms of mental sluggishness, rough and yellow fur, less shiny fur, inappetence, and thin and smelly excreta. These symptoms were consistent with the general manifestations of phlegm-dampness syndrome, indicating the success of modeling HUA with phlegm-dampness.

## Treatment of HUA with TCM

Presently, standard biomedical treatment for HUA mainly depends on drugs with active compounds such as benzbromarone (Zhao et al., 2022), probenecid (Granados, 2022), allopurinol (Rodevand et al., 2004), febuxostat (Chinchilla et al., 2016), and topiroxostat (Kario et al., 2021). These drugs have proven to be effective but are accompanied by side effects such as poor medication compliance and SUA rebound (Strilchuk, 2019). Therefore, clinicians should consider non-pharmacological dietary interventions before using these drugs (Liska, 2021). In addition to dietary interventions, TCM has become a choice for HUA treatment because of its mild but efficient therapeutic effect.

Based on dialectical thinking, TCM is guided by the “holistic view” and “prevention of disease” theory. It regulates all aspects of UA metabolism, highlighting the advantages of effects and multiple targets in the treatment of HUA. According to the TCM literature, HUA is treated by removing dampness and turbidity, strengthening the spleen and kidney, promoting blood circulation, eliminating blood stasis, *etc.* Therefore, high SUA levels are mainly regulated by damp-clearing, heat-clearing, rheumatism-clearing, and blood circulation-promoting drugs. Recent studies have shown that TCM plays a vital role in treating HUA by decreasing UA production, promoting UA excretion, inhibiting urate oxidase activity, regulating the expression of inflammatory factors, and improving the body’s self-regulation ability.

### Single-botanical drugs for treating HUA

This study used a single-botanical drug as the research object. The approach involved systematically sorting its mechanism of action on UA metabolism and providing new ideas for drug screening, prescription compatibility, and target determination in the treatment of HUA. The study further explored the therapeutic effect of single-botanical drugs on HUA and provided a theoretical basis for experimental research and clinical treatment of HUA (see Table 2).

**TABLE 2:** Treatment of HUA with TCM in animals.

TCM	TCM extract	Model animal	Modeling method	Dose of drug administered	Minimum active dose	Positive control drug	Intervention interval
*Cromidon plantaginis* (L.f.) Hilliard [Scrophulariaceae]	Alcohol extract	KM Mice	PO (400 mg/kg)	4.68, 2.34, and 1.17 g/kg	1.17 g/kg	Allopurinol (10 mg/kg)	7 d
*Polygonum cuspidatum* Siebold & Zucc [Polygonaceae]	Alcohol extract	SD Rat	PO (200 mg/kg) + adenine (100 mg/kg)	2.16, 1.08, and 0.54 g/kg	0.54 g/kg	Benzbromarone (11 mg/kg)	21 d
*Polygonum cuspidatum* Siebold & Zucc [Polygonaceae]	Alcohol extract	SD Rat	Adenine (200 mg/kg)	480 and 120 mg/kg	120 mg/kg	Allopurinol (5 mg/kg)	21 d
Ginkgo L. [Ginkgoaceae]	Alcohol extract	KM Mice	PO (400 mg/kg)	4.68, 2.34, and 1.17 g/kg	1.17 g/kg	Allopurinol (10 mg/kg)	7 d
Ginkgo L. [Ginkgoaceae]	Aqueous extract	SD Rat	Yeast (15 g/kg) + adenine (80 mg/kg)	13.2 and 6.6 g/kg	6.6 g/kg	Benzbromarone (20 mg/kg)	35 d
	Aqueous extract	SD Rat	10% fructose water	10, 7.5, and 5 g/kg	5 g/kg	Benzbromarone (20 mg/kg)	42 d
	Aqueous extract	French quails	Yeast	16.7,13.3, and 6.6 g/kg	6.6 g/kg	Benzbromarone (20 mg/kg)	60 d
Poria	Alcohol extract, Aqueous extract	KM Mice	PO (300 mg/kg) + hypoxanthine (500 mg/kg)	200, 100, and 50 mg/kg	50 mg/kg	Allopurinol (15 mg/kg); Benzbromarone (7.8 mg/kg)	7 d
	Aqueous extract	SD Rat	PO (100 mg/kg) + adenine (200 mg/kg)	2.0, 1.0, and 0.5 g/kg	0.5 g/kg	Allopurinol (10 mg/kg)	38 d
*Smilax glabra* Roxb. [Smilacaceae]	Alcohol extract	SD Rat	Yeast + adenine (50 mg/kg)	500, 300, and 100 mg/kg	100 mg/kg	Allopurinol (40 mg/kg)	35 d
*Pueraria montana* var. lobata (Willd.) Maesen & S.M.Almeida ex Sanjappa & Predeep [Fabaceae]	Aqueous extract, Alcohol extract	SD Rat	High-purine feed	5.25, 3.50, and 1.75 g/kg	1.75 g/kg	Allopurinol (10 mg/kg)	25 d
*Lonicera sempervirens* L. [Caprifoliaceae]	Alcohol extract	ICR Mice	PO (250 mg/kg)	500 and 300 mg/kg	300 mg/kg	Allopurinol (10 mg/kg)	No
*Portulaca quadrifida* L. [Portulacaceae]	Aqueous extract	ICR Mice	Hypoxanthine (0.3 g/kg) + PO (0.3 g/kg)	1.48 and 0.74 g/kg	0.74 g/kg	Allopurinol (20 mg/kg)	21 d
*Taraxacum* sect. *Taraxacum* F.H.Wigg. [Asteraceae]	Alcohol extract	Wistar Rat	PO (100 mg/kg) + hypoxanthine (500 mg/kg)	60, 30, and 15 g/kg	15 g/kg	Benzbromarone (20 mg/kg)	No
*Gardenia thunbergia* Thunb. [Rubiaceae]	Alcohol extract	KM Mice	PO (250 mg/kg)	556, 278, and 139 mg/kg		Allopurinol (5 mg/kg)	7 d
*Perilla frutescens* L. Britton [Lamiaceae]	Aqueous extract	KM Mice	Yeast (15 g/kg) + PO (200 mg/kg)	400 and 200 mg/kg	400 mg/kg	NO	28 d
Chrysanthemi Flos	Alcohol extract	SD Rat	PO (2 g/kg)	250, 100 mg/kg	100 mg/kg	Allopurinol (25 mg/kg)	21 d
*Chrysanthemum* × *morifolium* (Ramat.) Hemsl. [Asteraceae]	Aqueous extract	KM Mice	PO (250 mg/kg) + yeast feed (25%)	10, 5, 2.5 g/kg	10 g/kg	Allopurinol (40 mg/kg)	21 d
*Zingiber officinale* Roscoe [Zingiberaceae]	Alcohol extract	SD Rat	Yeast (5 g/kg) + PO (200 mg/kg)	50, 25, 5 mg/kg	5 mg/kg	Allopurinol (10 mg/kg)	28 d
*Dioscorea polystachya* Turcz. [Dioscoreaceae]	Alcohol extract	SD Rat	PO (250 g/kg)	480 mg/kg	480 mg/kg	NO	35 d
*Alpinia galanga* L.) Willd. [Zingiberaceae]	Aqueous extract	KM Mice	PO (500 mg/kg)	500, 250, and 125 mg/kg	125 mg/kg	Benzbromarone (20 mg/kg)	8 d
	Aqueous extract	KM Mice	Hypoxanthine (1,000 mg/kg)	500, 250, and 125 mg/kg	125 mg/kg	Allopurinol (40 mg/kg)	8 d

#### Damp-clearing drugs

*Cromidon plantaginis* (L.f.) Hilliard [Scrophulariaceae] (CPH)

*Plantaginis* is identified as a dry or fresh whole botanical drug of *Plantago asiatica* L. or *Plantago depressa* Willd. because of its functions of clearing heat, diuretic, expelling phlegm, cooling blood, and removing toxins. Its extracts *Plantaginis* inhibit the production of reactive oxide species (ROS), a kind of substance that can induce kidney injury, and downregulate the expression of URAT1 mRNA in the kidney, which improves kidney function and promotes UA excretion in mice (Zeng et al., 2013). CPH *Plantaginis* can also downregulate the expression of NLRP3, an apoptosis-related point-like protein (ASC), caspase-1, and the inflammatory factor IL-12 in renal tissue, thereby attenuating the inflammatory response (Zou et al., 2021). In addition, plantamajoside in CPH *Plantaginis* can decrease UA levels in PO-induced acute HUA mice by regulating renal UA transporter expression, TLR/MyD88/NF-κB pathways, and NLRP3 inflammatory pathways (Tingting, 2020).

*Polygonum cuspidatum* Siebold & Zucc. [Polygonaceae](PCSZ)

Polygoni Cuspidati *Polygonum cuspidatum Sieb*. is a traditional botanical drug with the functions of clearing heat, detoxifying, dispersing stasis, relieving pain, removing dampness, and eliminating jaundice. Based on a rat HUA model established using PO and adenine, Ye (2019) found that PCSZ significantly reduced SUA levels and protected the kidney. The possible mechanism of lowering SUA levels is inhibiting the reabsorption of UA by renal tubules by inhibiting the expression of GLUT9 and URAT1 mRNA and promoting UA excretion in the kidney by increasing the expression of OAT3 mRNA. In addition, polydatin can reduce the expression of inflammatory factors TNF-α, IL-1β, and IL-6 in serum, playing an anti-inflammatory role and alleviating inflammation-caused kidney injury (Ma et al., 2019).

*Dioscorea collettii* var. hypoglauca (Palib.) S.J.Pei & C.T.Ting [Dioscoreaceae] (DCVH)

*Dioscoreae Hypoglaucae*, *Dioscorea septemloba*, and *Dioscorea hypoglauca*, plays an important role in eliminating dampness and turbidity, dispelling pathogenic wind, and dredging channel blockage. In a rat model of HUA induced by PO and ethambutol, Chen et al. (2016a) found that the total saponins of Bixie could reduce SUA levels in a dose-dependent manner. DCVH could reduce the expression of URAT1 and decrease UA reabsorption. In contrast, it can upregulate the expression of OAT1, OAT3, and ABCG2 and promote the excretion of UA in the kidney. The ability of DCVH to decrease SUA by promoting UA excretion could be reflected by UA excretion indexes, such as 24 h UA excretion, UA excretion fraction, UA clearance rate, creatinine clearance rate, unit glomerular filtration UA excretion, and urinary UA content (Zhang et al., 2018).

Ginkgo L. [Ginkgoaceae], the dry leaves of *Ginkgo biloba* L., promote blood circulation, remove blood stasis, smooth collaterals, relieve pain, gather lungs and relieve asthma, remove turbidity, and reduce lipids. In addition, GF extract effectively reduced ICAM-1 and MCP-1 mRNA expression, decreased inflammatory factor release, and maintained glomerular endothelial cell stability (Cheng and Zhu, 2013). In summary, GF decreases SUA levels mainly by alleviating the inflammatory damage to the kidneys.

Cichorium intybus L. [Asteraceae]

*Cichorium intybus* is a perennial botanical drug belonging to the *Cichorium* genus of the Asteraceae family that causes diuresis detumescence and purging of the gallbladder and liver. It can significantly reduce UA levels through multiple pathways and target effects. Chicory reduces UA production by inhibiting the activities of key enzymes involved in UA metabolism. However, it can also increase UA excretion by inhibiting UA reabsorption in the kidney and regulating intestinal flora. Wang et al. (2017a) and Jin et al. (2018) used fructose-induced HUA in rats to study the uric-lowering mechanism of chicory. They found that decreased UA was associated with the downregulation of GLUT9 and ABCG2 and the upregulation of OAT1, OAT1-LIKE, and OAT3-LIKE (Wang et al., 2017b). Bian et al. (2020) chose quail fed with a high-purine diet as their research target. They found that chicory could recover intestinal flora distribution, reduce bacterial endotoxin levels in the cecal contents and peripheral blood, and decrease SUA levels.

Poria is a medicinal and edible fungus important in promoting water infiltration, strengthening the spleen, and soothing the heart. In HUA mice induced by hypoxanthine and PO, Liang et al. (2021) found that a poria extract decreased SUA levels and upregulated ABCG2 content. This protein lowers UA levels and protects the kidney. Wang et al. (2022) established an HUA rat model using adenine and PO to evaluate the anti-HUA efficacy of poria powder. They found that poria powder significantly improved renal function, glomerular atrophy, and renal tubule dilation in HUA rats. Poria powder also significantly improved the relative abundance of Bacteroidetes, Alistipes, and Parabacteroides in the intestinal tract and reduced the relative abundance of Firmicutes to regulate the intestinal flora. It was speculated that poria powder might promote UA excretion by regulating intestinal flora to lower UA and alleviate kidney injury.

#### Antipyretic drugs

*Smilax glabra* Roxb. [Smilacaceae]

Smilacis Glabrae is a rhizome of *Smilax glabra* Roxb. Studies have shown that SGR can effectively reduce SUA levels and protect kidneys through multi-target and multi-pathway synergistic functions in a PO-induced HUA mouse model. The mechanism has three aspects: one is that SGR can inhibit the activity of XO in the liver and subsequently inhibit the production of UA; one is that SGR can downregulate renal URAT1 and GLUT9 mRNA expression to promote renal UA excretion (Huang et al., 2019); the last one is that SGR can decrease the expression of IL-1β and TNF-α and alleviate the kidney injury caused by inflammation (Wang et al., 2019a).

*Gentiana quadrifaria* Blume [Gentianaceae]

*Gentianae* is a valuable TCM with functions such as clearing heat and dampness and purging the liver and gall. Gentiopicrin, the main active metabolite of gentian, significantly improved UA levels in PO-induced HUA mice. It regulates the expression of renal UA transporters, reduces the expression of GLUT9 and URAT1, and increases the expression of OAT1 and OAT3, thereby decreasing UA levels. Simultaneously, gentian inhibited the activation of NLRP3 inflammasomes produced because of excessive UA accumulation and relieved kidney inflammation (Au et al., 2021).

*Pueraria montana* var. lobata (Willd.) Maesen & S. M. Almeida ex Sanjappa & Predeep [Fabaceae]

Puerariae Lobatae dispels wind and dampness, activates blood circulation and collaterals, and invigorates the spleen with aromatics. In rats with HUA induced by yeast paste and PO, its extract showed anti-gouty arthritis and decreased SUA level function by inhibiting UA production and promoting UA excretion. Kudarin could significantly increase the activity of alkaline phosphatase and promote the expression of ABCG2 in HK-2 cells, thereby promoting UA excretion and reducing UA (Zhang et al., 2016).

*Lonicera sempervirens* L. [Caprifoliaceae]

Lonicerae Japonicae, because of its sweet taste, cold nature, and lung/stomach/heart tropism, has the functions of clearing heat and toxins, dispelling wind and heat, *etc.* Studies showed that honeysuckle can significantly inhibit XO activity and reduce SUA levels effectively in HUA mice induced by PO (Chien et al., 2009). Molecular docking showed that the binding energies of isochlorogenic acid B, isochlorogenic acid A, and chlorogenic acid or esculin with XO were higher than those with febuxostat, indicating higher inhibitory effects on XO. Both quercetin and isochlorogenic acid B have significant urate-lowering effects and inhibit XO activity in fructose-induced HAU rats (Peng, 2020).

Portulaca quadrifida L. [Portulacaceae]

*Portulaca oleracea* is a wild medicinal and edible plant important in clearing heat and dampness and detoxification and has anti-inflammatory, thirst relieving, and diuretic properties. Lv et al. (2022) established an HUA mouse model using hypoxanthine and PO stimulation. They found that purslane extract effectively reduced SUA levels in a dose-dependent manner in HUA mice but had little effect on urinary UA levels. The mechanism may be that the purslane extract reduces the activity of XO in the liver and further inhibits the production of UA in the body.

*Taraxacum* sect. *Taraxacum* F.H. Wigg. [Asteraceae]

*Taraxaci* is an Asteraceae plant that can clear heat and toxins, relieve swelling and eliminate mass, induce diuresis, and free strangury. Zou et al. (2020) established a rat model of acute HUA using a combination of PO and hypoxanthine. They administered dandelion extract intragastrically after 1 week. They found that different doses (60 g/kg, 30 g/kg, and 15 g/kg, respectively) of water, n-butanol, ethyl acetate, and petroleum ether extract significantly reduced SUA levels.

*Gardenia thunbergia* Thunb. [Rubiaceae]

*Gardenia jasminoides* protects the liver, benefits the gallbladder, relieves hypertension, maintains sedation, prevents bleeding, and reduces swelling. Hu et al. (2013) reported that geniposide, the main active metabolite in gardenia, could significantly reduce SUA levels and increase UA excretion to lower UA levels in the body by inhibiting the activity of XO in the liver and regulating the expression of mURAT1, mGLUT9, mOAT1, mOAT3, and mABCG2 in the kidneys of PO-induced HUA mice. In addition, gardenia extract reduced serum creatinine and urea levels and upregulated the expression of organic cationic/carnitine transporters, thereby repairing renal dysfunction in an HUA model (Meng et al., 2014)^.^

#### Drugs for relieving exterior disorder

*Perilla frutescens* (L.) Britton [Lamiaceae]

*Perilla frutescens* has analgesic, sedative, and detoxification properties. It can regulate SUA levels, promote renal UA excretion, and protect the renal tissue. Zhang et al. (2020) established an HUA mouse model by intragastrically administering yeast paste and PO. They found that the decrease in kidney index, UA, creatinine, and UA levels depended on the dose of *Perilla frutescens*.

*Chrysanthemum* × *morifolium* (Ramat.) Hemsl. [Asteraceae](CM) is an ornamental plant with medicinal value in dispelling wind-heat, tranquilizing the liver, improving eyesight, and clearing heat toxins. Shen (2019) reported that the inhibition of XO by the CM alcohol extract depended on the concentration of the CM alcohol extract, and the inhibition percentage of a high concentration of the CM alcohol extract on XO could reach 50%. Li et al. (2021b) screened three potential XO inhibitors from CM, genistein, luteolin, and apigenin, using surface plasmon resonance technology. Peng et al. (2019) established an HUA rat model by intragastric PO administration. They found that extracts of “Boju,” a kind of CM that is well-known in China, had an obvious UA-lowing effect and renal function protection ability by inhibiting XO activity and regulating the expression of renal UA transport-related proteins (ABCG2, URAT1, and GLUT9) and lipids. The extract also interfered with the pathological process of HUA by regulating biomarkers associated with the metabolism of tryptophan, sphingomyelin, glycerolipids, and arachidonic acid.

*Zingiber officinale* Roscoe [Zingiberaceae] (ZOR) relieves the exterior and dispels cold, warms the spleen and stomach, stops vomiting, warms the lungs, arrests cough, and removes toxins. Qi et al. (2011) found that a crude methanol extract of ZOR had an inhibitory effect on XO activity *in vitro*. However, only a dosage greater than 600 mg/kg demonstrated SUA-lowering ability and significantly inhibited XO activity in the liver.

#### Deficiency-tonifying drugs

Glycyrrhiza Tourn. ex L. [Fabaceae]

Glycyrrhizae invigorates the spleen, replenishes qi, moistens the lung, arrests cough, clears heat toxins, and reconciles multiple medications. Studies have shown that polysaccharides significantly inhibit XO activity at different doses, reducing SUA levels in rats (Guo, 2018). Wang et al. (2019b) found that glycyrrhizin, the main metabolite of licorice, significantly reduced blood UA levels and increased UA excretion in HUA mice. Glycyrrhizin downregulates the expression of OAT4 and URAT1 in the kidney tissues of HUA mice, thereby affecting UA metabolism and playing a role in UA reduction. Glycyrrhizin flavanol had a good ability to lower UA *in vitro*. It could improve recovery from oxidative stress injury in NRK-52E cells caused by high UA levels, possibly because of the inhibition of XO activity (Cao et al., 2022).

Sun et al. (2017) found that *Lycium barbarum* var. aurantiicarpum K.F. Ching [Solanaceae] extract at a medium dose (5 g/kg) and high dose (10 g/kg) significantly reduced the content of SUA and XO in HUA mice, indicating that the extract of Lycium barbarum maintained the efficacy of HUA treatment by reducing XO content to decrease UA production. Yu et al. (2020) established an HUA mouse model using PO. After treatment with *Lycium barbarum* polysaccharide, SUA level significantly decreased, whereas urinary UA level significantly increased. Correspondingly, they observed that OAT1 and OAT3 levels increased, and GLUT9 levels decreased in the kidney. Meanwhile, serum XO and liver XO activity decreased after treatment with *Lycium barbarum* polysaccharide.

*Gynostemma pentaphyllum* (Thunb.) Makino [Cucurbitaceae] is a cold-natured and bitter-tasting botanical drug that clears heat and toxins, arrests cough, clears lung heat, nourishes the heart for tranquilization, benefits qi, produces essence, and so on. Wu et al. (2020) found that gypenoside extract effectively inhibits the increase in SUA levels by inhibiting UA production and increasing UA solubility in body fluids, thereby protecting kidney function and promoting UA excretion. Pang et al. (2017) established an HUA animal model using a lipid emulsion; they found that the SUA, XOD, ADA, and XDH levels significantly decreased after gypenoside treatment. Simultaneously, URAT1 and GLUT9 expressions were downregulated, OAT1 expression was upregulated in the kidney, and the renal index and UA excretion fractions were increased.

##### Other drugs

Modern pharmacological studies have shown that *Dioscorea polystachya* Turcz. [Dioscoreaceae] (DPT) Dioscoreae Nipponice has anti-inflammatory and analgesic effects and is now widely used to treat inflammatory diseases. The total saponins in DPT can decrease UA production by inhibiting the activities of XO and ADA. Liu (2019) reported that total pangolin saponin could effectively adjust the mRNA and protein levels of URAT1, GLUT9, OAT1, and OAT3 and promote renal urate excretion in PO-induced HUA mice.

The total flavonoids in *Alpinia galanga* L.) Willd. [Zingiberaceae] *Alpinia officinarum* significantly reduced SUA levels by promoting UA excretion and inhibiting UA production in HUA mice. Xue et al. (Xue et al., 2017; Xue et al., 2018) found that galangal extract lowered SUA levels by inhibiting hepatic XO activity in both PO-induced HUA mice and hypoxanthine-induced HUA mice.

*Sanghuangporus* spp. (SS) are the fruiting bodies of Porophyllaceae. Studies have shown that both the polysaccharide and alcohol extracts of SS have inhibitory effects on the activity of XO to some extent, and the alcohol extract of SS has better UA-lowering ability than the crude polysaccharide of SS. The polysaccharide of SS showed an anti-competitive inhibition effect, whereas the alcohol extract showed non-competitive inhibition when interacting with XO (Li et al., 2022a).

*Nelumbo nucifera* Gaertn. [Nelumbonaceae] can reduce UA levels and improve renal function by regulating the expression of organic ion transporters in the kidneys during HUA treatment. It can also inhibit the activation of the TLR4/MyD88/NF-κB pathway and the NLRP3 inflammasome, relieving the renal inflammatory response (Wang et al., 2015). In addition, Liu (2019) showed that flavonoids can inhibit XO activity *in vitro*.

Presently, the ability of TCM to dissolve urate is insufficient to reach a level similar to that of urate oxidase. However, studies have demonstrated that some TCMs (such as *Cremastra appendiculata* (D.Don) Makino [Orchidaceae], *Atractylodes lancea* (Thunb.) DC. [Asteraceae], and *Rheum palmatum* L. [Polygonaceae]), can promote the dissolution of urate better than the 5% sodium bicarbonate solution commonly used in surgical lavage (Lin et al., 2022).

### TCM prescription for treating HUA

Tufuling granules remove turbidity, eliminate pathogens, remove dampness, and clear heat. HUA was induced with PO (100 mg/kg) by intraperitoneal injection and hypoxanthine (500 mg/kg) by oral gavage. The tufuling granule solution was administered by gavage at doses of 1 g/kg, 2 g/kg, and 4 g/kg, whereas the positive control group was administered febuxostat (4 mg/kg) by gavage. Medium and high doses of granules effectively reduced SUA levels in model rats. Tufuling granules inhibit XDH mRNA expression and reduce XO activity in the liver. Therefore, they can lower UA production directly or indirectly and are s usually used for the clinical treatment of HUA (Zhu, 2015).

An HUA model was established by combining yeast (15 g/kg), adenine (100 mg/kg), and potassium oxonate (300 mg/kg). Fan et al. (2017) found that ermiao pill could reduce UA production by inhibiting the activity of XO and reducing UA levels in HUA rats. In addition, Lv et al. (Lv et al., 2010) found that an ermiao pill and Huangbo water extract significantly reduced the expression of mURAT1 in the kidney of HUA mice, indicating that they could reduce UA levels by inhibiting the function of UA reabsorption transporters.

The classical Guizhi decoction effectively decreases the production of UA by inhibiting the activity of XO in the liver, promoting UA excretion by downregulating the protein levels of URAT1 and GLUT9 and upregulating the protein levels of ABCG2, OCT1, OCT2, OCTN1, and OCTN2 in the kidneys of PO-induced HUA mice. In other words, the Guizhi decoction reduced SUA levels and protected the kidneys (Tan, 2014).

Zuo et al. (2018) established an HUA mouse model (induced by hypoxanthine and PO) to investigate the effect of the erding decoction on reducing UA levels. They found that the erding decoction has anti-inflammatory and analgesic effects. It reduced UA levels by inhibiting URAT1 mRNA expression and increasing OAT3 mRNA expression. They also found that the 50% ethanol extract of erding granules could reduce UA levels and improve the pathological injury of the kidney by inhibiting the expression of inflammatory factors TNF-α, IL-1β, and IL-6 and regulating the expression of GLUT9 and OAT1 proteins.

Qiling formula granule, a clinical prescription, strengthens the spleen by benefitting qi, eliminates blood stasis, resolves turbidity, clears heat, and promotes diuresis. The HUA model was established by feeding mice 10% yeast meal combined with adenine (0.1 g/kg) and PO (0.1 g/kg). Studies have shown that qiling formula granules improve renal function and lipid metabolism in rats with HUA (Zhang and Xu, 2020). A possible mechanism is that qiling formula granules could inhibit the activity of XO in the liver, downregulate the mRNA level of URAT1 in the kidney, and upregulate the mRNA levels of OAT1 and OCT2. Therefore, they promoted UA excretion by reducing the reabsorption of renal urate and increasing its secretion.

Liang et al. (2016) modified Simiao powder and evaluated its efficacy in adenine- (100 mg/kg) and PO-induced (1.5 g/kg) HUA rats. They found that the modified Si-Miao-San powder had an obvious effect on reducing SUA levels and could significantly improve kidney injury caused by HUA, which might be related to the upregulation of URAT1 and GLUT9 protein expression and the downregulation of OAT1 protein expression in the kidney (Cao et al., 2021).

## Discussion and Conclusion

HUA is a metabolic disease caused by multiple factors. Establishing research models plays an important role in recapitulating HUA and developing a deeper understanding of the disease mechanism. In this review, we systematically discuss HUA evaluation model construction methods using cell or animal models by increasing the source of UA, inhibiting the activity of uricase, inhibiting the excretion of UA, knocking out genes, and destroying intestinal flora. However, cell models cannot fully replicate the pathogenesis of HUA *in vitro* because of the lack of a complex cellular microenvironment, and animal models face problems such as long experimental periods, high experimental costs, and species divergence. Although it is a key tool for studying the mechanism of HUA and drug development, the HUA model still has some problems and limitations regarding the effect stability and mechanism of HUA. Currently, most relatively mature and widely used animal models use a combination of several different modeling drugs that synergistically increase UA. The combination of UA precursors and UOX inhibitors has the advantages of rapidly increasing SUA levels, prolonging maintenance time, and reducing renal damage. Compared to animal models, cell models can be used for efficient, sensitive, and accurate screening of urate-lowering drugs at the cellular and molecular levels, especially for high-throughput drug screening. However, some shortcomings remain in constructing HUA cell models, such as the complex isolation of primary cells, highly differentiated cells, and single drug targets.

In summary, cell models cannot fully replicate the pathogenesis of HUA *in vitro* because of the lack of a complex cell microenvironment, and animal models face problems with long experimental periods, high experimental costs, and species divergence. Therefore, appropriate animal and cell models and methods should be selected according to the research purpose and experimental conditions. New alternative research techniques, such as organ-on-chips or organoids (Li et al., 2022b; Hou et al., 2022), should be considered to fully recapitulate the physiological and pathological characteristics of HUAs.

As HUA has become a common and high-risk metabolic disease, it is important to develop drugs for the prevention and treatment of HUA. Many studies have confirmed that TCM can treat HUA through multiple pathways and targets, with the advantages of few side effects. TCM can both reduce the level of UA and protect organs from damage, which has great research and development potential in the prevention and treatment of HUA, especially damp-clearing drugs. The pathogenesis of HUA is spleen deficiency and dampness excess. Damp-clearing drugs are based on instilling spleen and removing dampness and treat the spleen and kidney simultaneously, showing unique advantages among many TCMs.

In recent years, the mechanism of action of TCM in the treatment of HUA mainly plays a role in promoting UA excretion, inhibiting UA production, regulating gene expression, and affecting the expression of inflammatory mediators. UA metabolism is closely related to intestinal microflora. Therefore, taking UA intestinal metabolism as the entry point and intestinal microflora as the target, a future research direction is to systematically elaborate the mechanism of the interaction between TCM, intestinal microflora, and HUA. At present, there are many methods to improve intestinal flora disorders, such as probiotics, antibacterial drugs, and fecal microbiota transplantation technology. In the future, TCM intervention can be combined with these methods to improve HUA and promote the internationalization of TCM.

We focused on traditional HUA model construction methods and did not fully present all novel techniques, such as 3D HUA models. In addition, we emphasized the role of TCM in HUA treatment, with few chemical drugs available. This study had some limitations. However, a comprehensive introduction to the recent research progress of HUA in evaluation model construction and TCM intervention will undoubtedly provide a scientific reference for TCM-related HUA studies. In the future, TCM or multiple active compound combinations, together with a reasonable and reliable disease modeling method, will be an efficient pathway for HUA studies and provide a new reference and direction for the clinical treatment of HUA.
